# Work schedule characteristics associated with sleep disturbance among healthcare professionals in Europe and South Korea: a report from two cross-sectional surveys

**DOI:** 10.1186/s12912-022-00974-3

**Published:** 2022-07-18

**Authors:** Ari Min, Hye Chong Hong

**Affiliations:** Department of Nursing, Chung-Ang University, Seoul, South Korea

**Keywords:** Sleep, Shift work schedule, Health care professionals

## Abstract

**Background:**

Healthcare professionals worldwide are prone to sleep disturbance. Such sleep disturbance is associated with lower patient safety and quality of care. Therefore, this study aimed to identify the prevalence of sleep disturbance and determine the effect of work schedule characteristics on sleep disturbance among healthcare professionals in Europe and South Korea.

**Methods:**

We used the sixth European Working Conditions Survey and the fifth Korean Working Conditions Survey for analyses. The study included 2285 healthcare professionals aged 18–65 years. Work schedule characteristics included shift work, night work, working hours per week, long work hours (i.e., more than 10 hours per shift), quick return to work, having to come to work on short notice, and changes in work schedules. A multiple logistic regression analysis was performed.

**Results:**

The overall prevalence of sleep disturbance was 37.7%. The multivariate logistic regression model indicated that long work hours, quick return to, having to come to work on short notice, and changes in work schedules were significant factors associated with sleep disturbance among healthcare professionals.

**Conclusion:**

The findings revealed that several work schedule-related factors were associated with sleep disturbances in healthcare professionals in Europe and Korea. Institutions and policymakers should implement strategies and policies to reduce the unpredictability of work schedules to ensure an adequate rest period between shifts and to reduce sleep disturbances.

## Background

Unstable or irregular work schedules are “schedules in which the times of work vary, and workers have little or no control over that variability, either as individuals or through collective agreements” [1]. Approximately 10% of the labor force is assigned to unstable or irregular work schedules [2]. These unstable work schedules involve shift work, night shifts, rotating shifts, on-calls, quick return to work, having to come to work on short notice, and mandatory overtimes, and are prevalent in healthcare settings, as patient care is needed 24 hours a day, 365 days a year, to maintain continuity of care and ensure patient safety [1–3]. Irregular working schedules, including shift work, may significantly influence workers’ physical and mental health, as well as their job performance [4]. In particular, shift work is associated with accidents, obesity, coronary artery disease, stroke, cancer, diabetes [5], disruption in the circadian rhythm, and sleep disturbance [6]. However, there is a lack of evidence for the relationship between work schedules and sleep disturbance that could affect patient safety and quality of care of healthcare professionals.

Sleep disturbance is one of the most critical health issues worldwide [7]. The importance of sleep for maintaining health, safety, and quality of life is well documented in the literature [8]. Poor sleep quality and inadequate sleep are strong risk factors for adverse health outcomes, including cardiovascular disease, metabolic disorders, and cognitive dysfunction [9, 10]. Furthermore, decreased sleep quality and quantity are associated with accidents and decreased quality of life [11–13]. Insufficient or poor-quality sleep is associated with fatigue, leading to decreased alertness and slow reaction time, which markedly affects the function of healthcare workers, particularly those involved in patient care [6]. The Center for Disease Control and Prevention (CDC) reported that more than half of shift workers in health-care settings sleep fewer than 6 hours a day [14], which is far less than the amount of sleep recommended in medical guidelines (i.e., ≥7 h a day) [15].

Various work schedules are associated with sleep disturbances among workers in industries other than the healthcare industry. For example, working long hours in the week has been associated with difficulty falling asleep, frequent waking, non-refreshed sleep, and early awakening [16]. Shift length is associated with average sleep quality [17]. Night work is associated with difficulty falling asleep, difficulty waking, and not feeling rested [18]; a quick return to work is associated with insomnia and excessive sleepiness. Moreover, unstable work schedules or short advance notices of schedule changes are significant predictors of workers’ sleep disturbance [19].

We also note that sleep disturbance is a major public health issue in the general population [7, 18] and may be affected by various work schedules of healthcare professionals. Healthcare professionals commonly have unstable or irregular work schedules. Sleep disturbance can affect not only their own well-being but also patient safety; therefore, studies determining the effect of various work schedules on sleep disturbance in this population are imperative. The purpose of the current study was to identify the prevalence of sleep disturbance and to determine the effects of work schedule characteristics on sleep disturbance among healthcare professionals in European countries and in South Korea, using two national working condition surveys. Our study aims to provide important data for policy development related to work schedules both nationally and globally.

## Methods

### Data source and sample

The present study was a secondary analysis of data obtained using the sixth European Working Conditions Survey (EWCS) and the fifth Korean Working Conditions Survey (KWCS), which were the most up-to-date datasets at the time of conducting this study. The EWCS data were collected between February and December of 2015, and the KWCS data were collected between July and November of 2017. Both surveys are nationally representative. The KWCS was developed by benchmarking the EWCS. The surveys employed a cross-sectional design. Detailed information about these surveys is available on the relevant websites. Ethical review and approval for the analysis of these secondary data were waived by the Institutional Review Board of the first author’s university.

The sixth EWCS comprised data from 35 European countries and had 43,850 participants, and the fifth KWCS included 50,205 participants from Korea. In the present study, we restricted the eligible participants to healthcare professionals aged 18–65 years. Healthcare professionals were defined using the International Standard Classification of Occupations code for the EWCS and the codes of the Korean Standard Classification of Occupation and Korean Standard Industrial Classification for the KWCS. After excluding non-healthcare professionals, we also excluded participants over 65 years, as workers in Korea commonly retire at this age (28 participants in the EWCS and two participants in the KWCS were excluded). Finally, 2285 participants (1320 participants in the EWCS and 965 participants in the KWCS) were included in this study.

### Measures

#### Sleep disturbance

Sleep disturbance was measured by asking the participants if they experienced the following three sleep-related problems over the last 12 months: (1) difficulty falling asleep, (2) waking up from sleep repeatedly, and (3) waking up feeling exhausted and fatigued. There were five possible responses to each question: “daily,” “several times a week,” “several times a month,” “not very often,” and “never.” The “daily,” “several times a week,” or “several times a month” responses were categorized as indicating a sleep problem, and “not very often” and “never” responses were categorized as indicating an absence of a sleep problem. In this study, participants categorized as having at least one sleep-related problem were considered as experiencing sleep disturbance.

#### Work schedule characteristics

The study included seven work schedule characteristics: shift work, night work, working hours per week, long work hours, quick return to work, having to come to work on short notice, and changes in work schedule. Participants who answered that they worked shifts were categorized as shift workers. We categorized participants as night-shift workers if they worked at least 2 hours between 10 p.m. and 5 a.m. during a month. Working hours per week were assessed by asking workers how many hours they usually worked per week. The number of hours was categorized as < 40, 40–52, and > 52 hours according to the Labor Standards Acts in Korea, which states that employees can work from 40 to a maximum of 52 hours per week [20]. Long work hours refer to working more than 10 hours on any single day of the month. Quick return to work was measured if participants had less than 11 hours between shifts (between the end of one working day and the start of the next working day) at least once in the previous month. Having to come to work on short notice was assessed by asking the question, “How often have you been requested to come to work at short notice?” Participants who answered “daily,” “several times a week,” or “several times a month” were categorized as having experienced coming to work on short notice, and those who answered “not very often” or “never” were categorized as not having had this experience. Changes in work schedule were assessed by asking the participants if changes to their working time arrangements occurred and how much advance notice they received regarding the changes. Participants who answered “yes” and responded that this occurred on “the same day,” “the day before,” “several days in advance,” or “several weeks in advance” were defined as having experienced changes in their work schedule.

#### Covariates

Age, sex, and educational level were included as covariates. Age was categorized into groups < 30, 30–39, 40–49, 50–59, and ≥ 60 years. The mean age of European participants was 43.29 years (standard deviation [SD] = 11.23 years) and of Korean participants was 38.41 years (SD = 9.67 years). Most participants were female (78.0% in Europe, 86.5% in Korea). Educational level was categorized as an associate degree, a bachelor’s degree, and a master’s or higher degree. Approximately 76% of the European participants had an associate degree, while approximately half of the Korean participants had a bachelor’s degree or higher (44.9% had a bachelor’s degree, and 4.3% had a master’s or higher degree).

### Data analysis

Descriptive statistics, including frequency and percentage, were used to describe the characteristics of the study participants. The prevalence of sleep disturbance according to work schedule characteristics was analyzed using the chi-squared test. A series of univariate and multiple logistic regression analyses were performed to examine the effect of work schedule characteristics on sleep disturbance among healthcare professionals after controlling for age, sex, and educational level. First, univariate logistic regression models were tested, and then a forward stepwise multiple logistic regression analysis was performed by including work schedule characteristics that yielded a *p*-value < .05. As a supplementary analysis, we investigated the effects of work schedule characteristics on presenteeism according to EWCS and KWCS separately using multivariate logistic regression models controlling for age, sex, and educational level. Data were analyzed using STATA statistical software (v.15.1; StataCorp LP, College Station, TX, USA). A *p-*value < .05 indicated statistical significance. We determined 95% confidence intervals (CI).

## Results

Table 1 shows the characteristics of the study participants. Shift workers, particularly night shift workers, comprised more participants in Europe than in Korea. The majority of the participants in Europe worked for less than 40 hours per week, even though they worked long hours, while more than half of the participants in Korea worked over 40 hours per week and only 8.2% had long work hours. More European participants experienced a quick return to work than Korean participants. In Europe, a higher number of participants reported coming to work on short notice, and experiencing work schedule changes compared with Korean participants.

**Table 1:** Descriptive characteristics of the study population (*N* = 2285)

Characteristics	Categories	EWCS (*n* = 1320)	KWCS (*n* = 965)	*p-*value
Age (years)	< 30	197 (14.9)	208 (21.6)	<.001
	30–39	322 (24.4)	333 (34.5)	
	40–49	355 (26.9)	279 (28.9)	
	50–59	350 (26.5)	137 (14.2)	
	≥ 60	96 (7.3)	8 (0.8)	
Gender	Male	290 (22.0)	130 (13.5)	<.001
	Female	1030 (78.0)	835 (86.5)	
Educational level	Associate degree	977 (76.4)	490 (50.8)	<.001
	Bachelor’s degree	103 (8.0)	433 (44.9)	
	Master’s or higher	199 (15.6)	42 (4.3)	
Shift work	Yes	562 (42.6)	230 (23.8)	<.001
	No	756 (57.4)	735 (76.2)	
Night work	Yes	493 (37.9)	175 (18.1)	<.001
	No	806 (62.1)	790 (81.9)	
Working hours per week	< 40	973 (75.1)	456 (47.3)	<.001
	40–52	244 (18.8)	452 (46.9)	
	> 52	79 (6.1)	56 (5.8)	
Long work hours (> 10 h)	Yes	539 (42.1)	79 (8.2)	<.001
	No	741 (57.9)	886 (91.8)	
Quick return (within < 11 h)	Yes	399 (30.5)	38 (3.9)	<.001
	No	909 (69.5)	926 (96.1)	
Work on short notice	Yes	660 (50.3)	183 (19.0)	<.001
	No	653 (49.7)	779 (81.0)	
Changes in work schedule	Yes	361 (40.4)	211 (23.3)	<.001
	No	533 (59.6)	693 (76.7)	

*Abbreviations*: *EWCS* European Working Conditions Survey, *KWCS* Korean Working Conditions Survey

Table 2 shows the prevalence of sleep disturbance according to the participants’ characteristics. The overall prevalence of sleep disturbance was 37.7% (*n* = 861); the prevalence rate was higher in the European participants (50.0%) than in the Korean participants (20.8%). Sleep disturbances were more prevalent among shift workers, particularly participants who worked night shifts, over 52 hours per week, and long work hours. Healthcare professionals who experienced a quick return to work, worked on short notice, and with short-notice changes in their work schedule, experienced more prevalent sleep disturbance than those who did not experience these work conditions.

**Table 2:** Prevalence of Sleep Disturbance among Healthcare Professionals (*N* = 2285)

Characteristics	Total	Sleep disturbance		*p-*value
		Yes	No	
		n (%)		
Overall				
EWCS	1320	660 (50.0)	660 (50.0)	<.001
KWCS	965	201 (20.8)	764 (79.2)	
Shift work				
Yes	792	332 (41.9)	460 (58.1)	.003
No	1491	529 (35.5)	962 (64.5)	
Night work				
Yes	668	295 (44.2)	3733 (55.8)	<.001
No	1596	557 (34.9)	1039 (65.1)	
Working hours per week				
< 40	1429	586 (41.0)	843 (59.0)	<.001
40–52	696	201 (28.9)	495 (71.1)	
> 52	135	62 (45.9)	73 (54.1)	
Long work hours (> 10 h)				
Yes	618	322 (52.1)	296 (47.9)	<.001
No	1627	521 (32.0)	1106 (68.0)	
Quick return to work (< 11 h)				
Yes	437	240 (54.9)	197 (45.1)	<.001
No	1835	615 (33.5)	1220 (66.5)	
Work on short notice				
Yes	843	388 (46.0)	455 (54.0)	<.001
No	1432	470 (32.8)	962 (67.2)	
Changes in work schedule				
Yes	572	252 (44.1)	320 (55.9)	<.001
No	1226	365 (29.8)	861 (70.2)	

*Abbreviations*: *EWCS* European Working Conditions Survey, *KWCS* Korean Working Conditions Survey

**Fig. 1:**
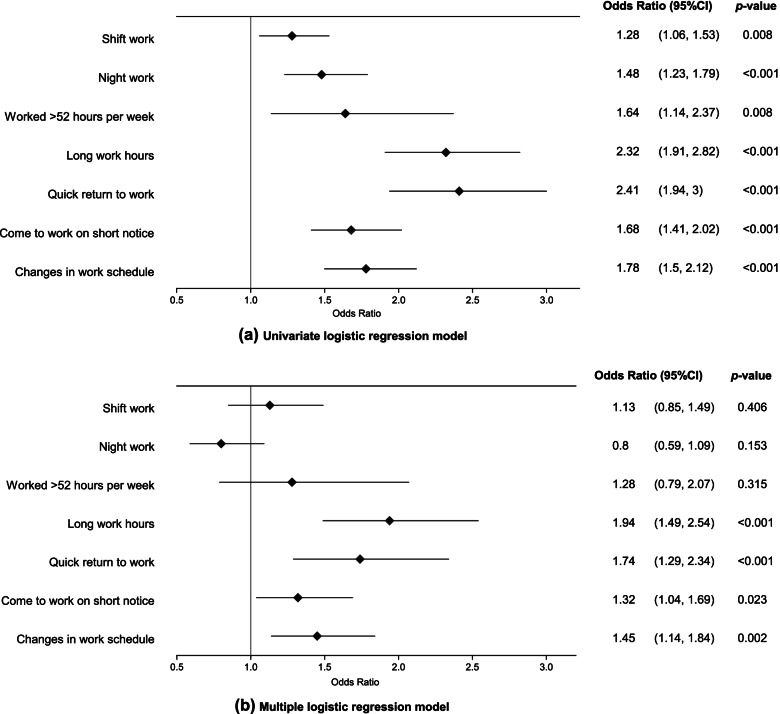
The odds ratios of work schedule characteristics for causing sleep disturbance among healthcare professionals. **a** Univariate logistic regression model. **b** Forward stepwise multiple logistic regression analysis (*p* < .05 for inclusion). All models are adjusted by age, sex, and education level. CI: confidence interval

Figure 1 shows the work schedule characteristics associated with sleep disturbance. Univariate logistic regression models revealed that shift work (odds ratio [OR] = 1.28, 95% CI = 1.06–1.53), night work (OR = 1.48, 95% CI = 1.23–1.79), working > 52 hours/week (OR = 1.64, 95% CI = 1.14–2.37), long work hours (OR = 2.32, 95% CI = 1.91–2.82), quick return to work (OR = 2.41, 95% CI = 1.94–3.00), having to come to work on short notice (OR = 1.68, 95% CI = 1.41–2.02), and changes in work schedule (OR = 1.79, 95% CI = 1.50–2.12) were significant factors associated with sleep disturbance (Fig. 1a). In the stepwise multiple logistic regression model, long work hours (OR = 1.94, 95% CI = 1.49–2.54), quick return to work (OR = 1.74, 95% CI = 1.29–2.34), having to come to work on short notice (OR = 1.32, 95% CI = 1.04–1.69), and changes in work schedule (OR = 1.45, 95% CI = 1.14–1.84) remained significant (Fig. 1b).

Table 3 shows the work schedule characteristics associated with sleep disturbance by study population (i.e., results were provided separately for healthcare professionals in Europe and in Korea). Working > 52 hours/week (OR = 1.65, 95% CI = 1.01–2.72), long work hours (OR = 1.52, 95% CI = 1.21–1.92), and quick return to work (OR = 1.59, 95% CI = 1.25–2.03) were statistically significantly associated with sleep disturbance in European participants. In Korean participants, working > 52 hours/week (OR = 1.03, 95% CI = 1.14–2.37) and changes in work schedule (OR = 1.79, 95% CI = 1.50–2.12) were statistically significantly associated with sleep disturbance.

**Table 3:** The odds ratios of work schedule characteristics on sleep problems among healthcare professionals (*N* = 2285)

Characteristics	EWCS			KWCS		
	Odds ratio	95% CI	*p*-value	Odds ratio	95% CI	*p*-value
Shift work						
Yes	0.88	0.69–1.10	0.258	1.27	0.89–1.82	0.186
No	1.00			1.00		
Night work						
Yes	1.17	0.93–1.47	0.185	0.93	0.61–1.40	0.713
No	1.00			1.00		
Worked > 52 hrs hours per week						
Yes	1.65	1.01–2.72	0.048	1.85	1.03–3.33	0.039
No	1.00			1.00		
Long work hours (> 10 hrs)						
Yes	1.52	1.21–1.92	< .001	1.60	0.95–2.67	0.074
No	1.00			1.00		
Quick return to work (< 11 hrs)						
Yes	1.59	1.25–2.03	< .001	1.77	0.87–3.58	0.113
No	1.00			1.00		
Work on short notice						
Yes	1.14	0.91–1.42	0.247	1.36	0.93–1.99	0.108
No	1.00			1.00		
Changes in work schedule						
Yes	1.29	0.99–1.71	0.063	2.09	1.47–2.98	< .001
No	1.00			1.00		

*Abbreviations*: *CI* confidence interval, *EWCS* European Working Conditions Survey, *KWCS* Korean Working Conditions Survey

All models were adjusted for age, sex, and education level

## Discussion

The study aimed to identify the prevalence of sleep disturbance among healthcare professionals in Europe and Korea and to determine the effect of work schedule characteristics on sleep disturbance among these individuals by using two national working condition surveys. Addressing the sleep disturbance of healthcare professionals is of paramount importance as sleep disturbance is associated with fatigue and sickness presenteeism, with consequential ill- health, which in turn could affect the quality of patient care and optimal performance among healthcare professionals [21–25]. Previous studies have reported that one-third of the general population experienced insomnia or sleep disturbances [26, 27]. In our study, we found that approximately 37% of the healthcare professionals experienced sleep disturbances. In other studies, 43–50% of healthcare professionals reported sleep disturbances [28–30]. The discrepancy in the prevalence of sleep disturbances among healthcare professionals between studies may be due to differences in the definitions and measurements of sleep disturbances. There is no consensus on defining sleep disturbance or insomnia, and their definitions vary greatly depending on whether studies include either or both the quantity and quality of sleep [27]. Furthermore, the difference in the prevalence of sleep disturbances may be due to different instruments or methods used to measure them.

Although the prevalence of sleep disturbance in our study participants and the general population were similar, a difference between shift and non-shift workers was observed. Notably, sleep disturbances were more prevalent among shift workers, conforming to previously published findings [31–33]. Shift work disrupts the circadian rhythm and regulation of sleep [34] and thus affects both the quantity and quality of sleep. We also found that working a night shift was associated with sleep disturbances. This result was consistent with previous findings in night shift workers, including healthcare professionals [35–37]. Night shift work interferes with circadian and normal biological rhythms and hormones, thus leading to poor sleep quality, shortened and disrupted sleep, and excessive sleepiness while awake [38].

This study’s findings revealed that a majority of the healthcare professionals in Europe worked less than 40 hours per week, whereas more than half of the healthcare professionals worked over 40 hours per week in Korea. In particular, approximately 10% of Korean healthcare professionals worked more than 52 hours per week. Given that nurses comprise the largest sector of the healthcare profession, these findings are similar to those of previous studies, which reported that Korean nurses worked an average of 47 hours per week, with approximately 12% of nurses working over 52 hours per week [39]. Several factors may contribute to the long working hours per week among healthcare professionals in Korea. Korea has fewer physicians per person than other members of the Organization for Economic Cooperation and Development, at 2.2 physicians per 1000 people, compared to 4.4 in Norway and 4.1 in Germany, Sweden, and Switzerland [40]. Moreover, the nurse-to-patient ratio is 14.2 in Korea, which is markedly higher than the ratio of 8.6 in England [41, 42]. These high healthcare professional-to-patient ratios may contribute to the overtime and long work hours in Korea, which may be an important contributor to sleep disturbance [16] and adverse patient outcomes [43, 44]. The Labor Standards Act of Korea states that workers can work up to 40 hours per week and work up to 52 hours, including overtime, when mutually agreed [20]. However, healthcare professionals have been an exception to this act, and there are no current regulations for limiting overtime work or punishment for violating the 40 hours per week work regulation. Our results provide a preliminary evidence base for reducing total working hours and for developing stronger mandatory overtime regulations with the aim of mitigating sleep disturbances among healthcare professionals, thereby ensuring improved worker health and a higher quality of care for patients. However, we strongly recommend a follow-up longitudinal study examining the potential causal relationship between total working hours and sleep disturbance in future research. Moreover, there is a need for research examining the working environment in each healthcare professional category (physicians, nurses, nurse aides, and ancillary staff) as no statistics are available for specific healthcare professional categories.

The multiple logistic regression model employed in this study demonstrated that long work hours, quick return to work, having to come to work on short notice, and changes in one’s work schedule were each statistically significantly associated with sleep disturbance among the surveyed healthcare professionals. Moreover, in our supplementary analysis, we found that total working hours per week was a statistically significant predictor for sleep problems in both European countries and in Korea, which was consistent with the results of previous studies [45, 46]. We note that the results in European countries were consistent with previous findings demonstrating that long work shifts (> 12 h), long work hours per week, and a quick return to work were associated with increased sleepiness (which can lead to adverse outcomes for healthcare workers and for patients) [25, 47, 48]. In addition, long working hours have been shown to contribute to inadequate recovery between shifts, which can both lead to and exacerbate sleep disturbance [49]. Moreover, quick return to work is more prevalent among shift workers, including healthcare professionals, and could contribute substantially to the poor sleep quality/quantity and exhaustion evidenced among nurses [21, 50], as observed in our study. As compared to Korea, long work hours and quick return to work were more prevalent and had a significant impact on sleep disturbance among healthcare professionals in European countries. These findings indicate that policy makers and institutions should pay more attention to limiting long work hours and quick returns that could alter quality of care and patient safety and that this is especially relevant to healthcare professionals in European countries.

We also found that a variable work schedule was a statistically significant factor contributing to sleep disturbance in Korean healthcare professionals. A previous study reported that unstable work schedules are also associated with sleep disturbance in service workers [19]. When regular routines are not maintained due to work schedule changes, the irregularity results in sleep disturbance, as the body cannot adjust to the new schedules, resulting in additional health problems [51–53]. In fact, previous studies have indicated that a work schedule that provides workers with 2 weeks’ advance notice was shown to improve worker health and sleep quality [54]. Therefore, managers and supervisors may need to consider providing work schedules in a more timely manner as well as expending more effort on implementing effective strategies to maintain stability in work schedules, as informed by the current evidence base. However, there has been little research on the relationship between unstable schedules with sleep disturbance; thus, further studies are warranted among healthcare professionals.

### Limitations

The causality between sleep disturbance and work schedules cannot be assumed; thus, the results should be interpreted with caution. Although the data were derived from nationally representative samples, the proportion of each category of healthcare professionals was not differentiated in the datasets. Further studies should examine and compare sleep disturbance and its associated factors within categories of healthcare professionals. Moreover, in the present study, sleep disturbance was measured with three questions, which may not have adequately captured sleep quantity and quality and various aspects of sleep disturbances among the healthcare professionals. Thus, studies using measures incorporating the multidimensional nature of sleep disturbance or objective measures, such as actigraphy, should be used in future studies. Additionally, unified measures are needed to compare the prevalence of sleep disturbance and its associated factors across studies. Finally, we may not have controlled for all possible confounding variables that may have influenced sleep since this study was a secondary data analysis.

## Conclusion

The findings of our study showed that more than one-third of the healthcare professionals experience sleep disturbance, and several work schedule-related factors such as long work hours, quick return, having to come to work on short notice, and changes in work schedules were associated with sleep disturbances in European countries and Korea. Therefore, policymakers and administrators should pay close attention to these work schedule characteristics and develop policies that limit long work hours, unstable work schedules and guarantee adequate recovery between work days. There is also a great need to strictly monitor factors contributing to long work hours, such as overtime and healthcare professional to patient ratios, and to penalize hospitals or healthcare institutions who fail to comply. We also recommend multi country studies employing a longitudinal design to confirm the causality between work schedule characteristics and sleep disturbance.

## Data Availability

The data that support the findings of this study for the European Working Conditions Survey are openly available at https://www.eurofound.europa.eu/data/european-working-conditions-survey (10.5255/UKDA-SN-8098-4). The data that support the findings of this study for the Korean Working Conditions Survey are open available at at https://oshri.kosha.or.kr/oshri/researchField/workingEnvironmentSurvey.do.

## Abbreviations

EWCS: European Working Conditions Survey
KWCS: Korean Working Conditions Survey
